# A TME-activated nano-catalyst for triple synergistic therapy of colorectal cancer

**DOI:** 10.1038/s41598-024-53334-3

**Published:** 2024-02-09

**Authors:** Qiang Liu, Yurong Xiang, Qiang Yu, Quan Lv, Zheng Xiang

**Affiliations:** 1 Department of Gastrointestinal Surgery, The First Affiliated Hospital of Chongqing Medical University Chongqing, Chongqing, China; 2 Chongqing Key Laboratory of Department of General Surgery, The First Affiliated Hospital of Chongqing Medical University Chongqing, Chongqing, China; 3 Department of Hepatobiliary Surgery, Suining First People’s Hospital, Suining, China

**Keywords:** Cancer therapy, Drug delivery

## Abstract

Colorectal cancer cells are highly heterogeneous and exhibit various drug resistances, making personalized treatment necessary. This typically involves a combination of different treatment modalities such as surgery, radiation, and chemotherapy to increase patient survival. Inspired by this, synergistic therapy, which combines multiple modalities into a single nanotherapeutic drug, shows promise in treating cancer. In this study, a nano-catalyst based on calcium peroxide (CaO_2_) and the chemotherapeutic drug doxorubicin hydrochloride (DOX) co-loaded into HPB nanoparticles (HPB@CaO_2_/DOX-PAA) was developed to achieve synergistic cancer treatment through chemodynamic/chemo/photothermal (CDT/CT/PTT) mechanisms. After being endocytosed by cancer cells, the nano-catalyst decomposes, releasing cargo. During near-infrared light irradiation, HPB induces a photothermal effect, DOX exhibits significant RNA and DNA destruction capabilities, meanwhile CaO_2_ produces a large amount of H_2_O_2_ in the moderately acidic TME, which combines with Fe^2+^ ions derived from HPB to form the highly toxic •OH in a Fenton-like reaction, enhancing the chemodynamic treatment. Assays conducted ex vivo and in vivo have exhibited the efficacy of this triple synergistic therapeutic regimen, indicating its potential clinical application.

## Introduction

Each year, nearly 900,000 people around the world lose their lives due to colorectal cancer, which is the second deadliest type of cancer^1^. It is estimated that by 2030, there will be over 2.2 million new cases of colorectal cancer globally, resulting in 1.1 million fatalities^2^. Unfortunately, patients with advanced cancer and distant metastasis often do not have the option of surgery^3^, while chemotherapy and radiotherapy can have significant side effects like myelosuppression, neurotoxicity, and cardiotoxicity, which can limit their clinical use^4^. Despite this, surgery, chemotherapy, and radiotherapy are still the primary clinical treatments for colorectal cancer^5^. In recent years, there has been a lot of interest in new cancer treatments such as chemodynamic therapy (CDT)^6^, photothermal therapy (PTT)^7^, photodynamic therapy (PDT)^8^, starvation therapy^9^, and gas therapy^10^ because of their unique approaches to fight cancer. Combining different therapies in a synergistic way often leads to better therapeutic outcomes than using a single therapy alone^11–13^. This is because it allows for the benefits of multiple therapies to work together to kill cancer cells while minimizing harm to healthy tissues. As a result, researchers are increasingly focusing on developing and testing synergistic therapies for colorectal cancer.

Chemodynamic therapy (CDT) is a novel cancer treatment that uses the Fenton reaction or a similar reaction mechanism within the body^14,15^. This process involves an intratumoral reaction where hydrogen peroxide (H_2_O_2_) breaks down into hydroxyl radicals (•OH) with the help of metal catalysts like Fe, Mn, Cu, Co, etc. The •OH radicals cause oxidative damage to lipids, proteins, and DNA, leading to cancer cell death. However, low levels of H_2_O_2_ in tumor cells (50 × 10^6^–100 × 10^6^ mmol/L) can significantly reduce the efficiency of the Fenton reaction, making it less effective^16–18^. To increase the concentration of H_2_O_2_ within tumor cells, exogenous delivery of H_2_O_2_ is required. Calcium peroxide (CaO_2_), which is one of the metal peroxides, can release calcium ions and H_2_O_2_ in the acidic environment of a tumor microenvironment (TME). It can also slowly release O_2_ when exposed to water or heat^19^. Under the action of ferrous ions, the released H_2_O_2_ is transformed into the extremely poisonous •OH^20^, and the resulting O_2_ enhances the hypoxic tumor environment and boosts the sensitivity of chemotherapeutic medications like DOX^21^. Moreover, ongoing calcium ion build up in cancer cells results in intracellular calcification, which kills the cancer cells^22^.

Combining chemodynamic treatment (CDT), photothermal therapy (PTT), and chemotherapy (CT) has been identified as a promising strategy for enhancing the effectiveness of anticancer therapy^23^. PTT is a non-invasive tumor therapy in which, when exposed to laser light, a photothermal agent (PTA) transforms light energy into heat energy, raising tumor temperature to 41–47 °C and resulting in cell death^24^. In addition to directly harming cancer cells, heat energy can change tumor microenvironment, reducing hypoxia in most solid tumors^25^ and accelerating the production of ROS (such as •OH), which can improve the efficacy of CDT and offer more potent anticancer therapy^26^. Prussian blue (PB) is a member of the metal–organic framework (MOF), composed of two iron centers (ferric Fe(III) and ferrous Fe(II)) that are octahedrally coordinated. Ferric ions are linked to the nitrogen atoms of cyanides, while the carbon atoms of cyanides concatenate the ferrous ions. PB was approved by the US Food and Drug Administration (FDA) in 2003 as an antidote for the treatment of radioactive element poisoning, such as thallium and cesium^27^. PB also has high photothermal conversion efficiency (PCE) and excellent photothermal stability^28^, making it an ideal PTA. PB can be acid-etched to create hollow Prussian blue (HPB), which possesses the same photothermal properties as PB but also has imaging capabilities, multi-enzyme activity, high specific surface area, surface modifiability, controlled drug release, biocompatibility and biodegradability, and good stability. Due to these advantages, HPB is frequently employed in biomedicine^29^. Furthermore, the pharmacokinetics and mechanism of action of the chemotherapeutic medication doxorubicin hydrochloride (DOX) have been extensively studied, and with the assistance of NADP oxidase and under aerobic circumstances, DOX increases ROS generation, furthering irreversible oxidative damage to cancer cells^30^. Moreover, polyacrylic acid (PAA) helps HPB complete the pH-responsive release of the loaded medication owing to its pH-dependent swelling ratio when exposed to acidic TME. Therefore, it is expected that HPB with PAA coating will prevent CaO_2_ and DOX from decomposing before aggregating at the tumor site^31^.

**Figure 1:**
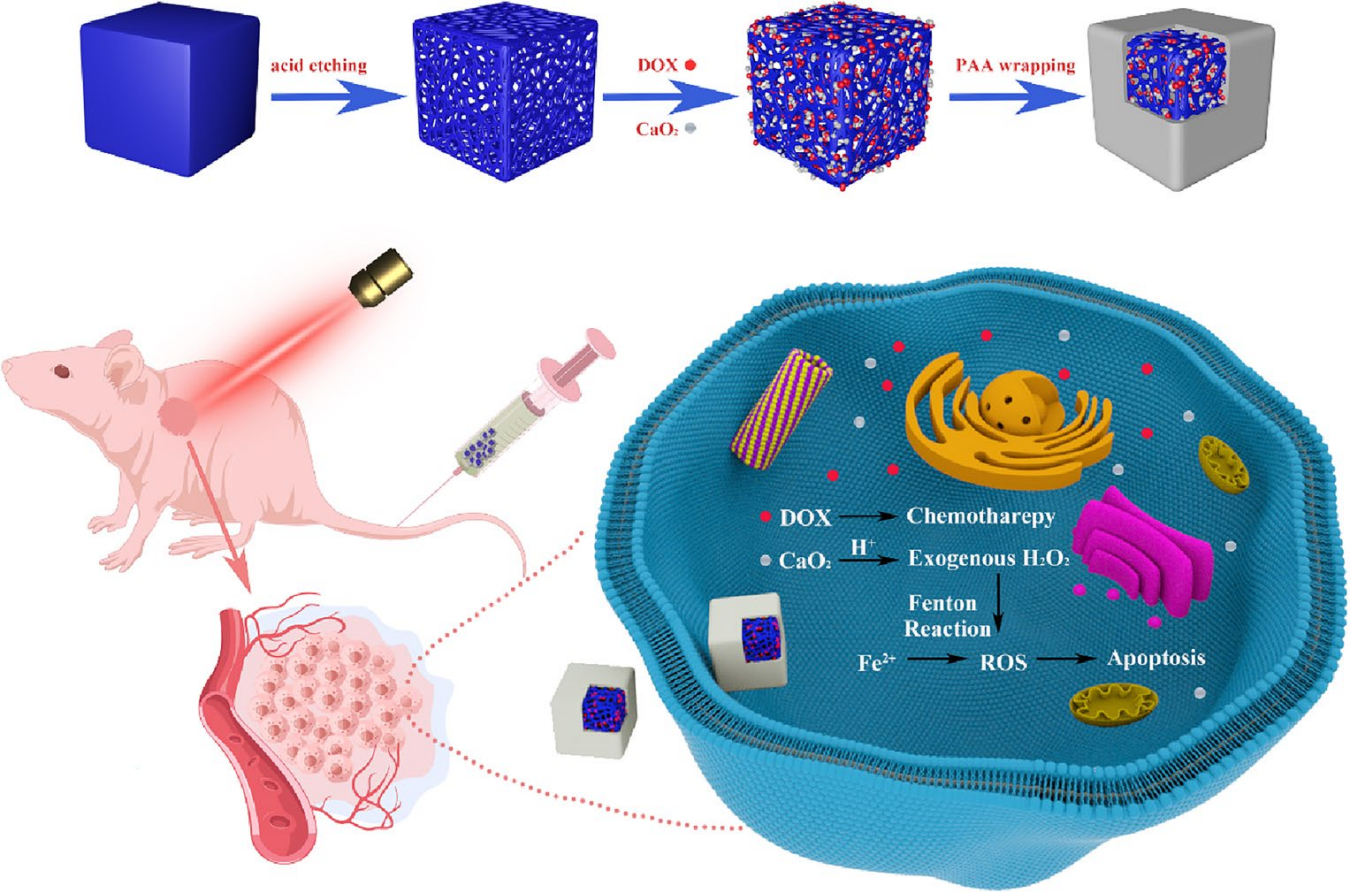
Synthesis procedure of PCDP nano-catalysts and multi-modal therapeutic mechanism in cancer treatment.

Herein, we designed a synergistic anti-tumor treatment mode combining CDT/CT/PTT, which has tumor specificity and therapeutic effect. As shown in Fig. 1, hollow Prussian blue nanoparticles (HPB) were synthesized based on previous reports, then loaded with DOX and CaO_2_, and coated with polyacrylic acid (PAA) to avoid drug leakage, finally forming a multifunctional nano-catalyst HPB@CaO_2_/DOX-PAA (PCDP). When PCDP enters the bloodstream, accumulates in cancer tissue through enhanced permeation retention effect (EPR) and is absorbed by cancer cells, the PAA on the surface of PCDP decomposes and releases chemotherapy drugs DOX and CaO_2_. CaO_2_ generates a large amount of H_2_O_2_ under acidic conditions, and H_2_O_2_ generates highly toxic •OH via Fenton reaction to achieve effective chemodynamic therapy (CDT) under the catalysis of ferrous ions. HPB not only serves as a carrier, but also can provide ferric Fe(III) and ferrous Fe(II). It can also play a photothermal (PTT) role under 808 nm near-infrared light (NIR) irradiation. Simultaneously, PTT promotes CDT efficiency, ameliorates cancer tissue hypoxia, and enhances DOX chemotherapy effect. Compared to the single treatment mode, this synergistic treatment method offers a higher therapeutic impact.

## Experimental section

### Chemicals and agents

All reagents and solvents (analytical grade) were used as received from commercial sources unless otherwise indicated. K_3_[Fe(CN)_6_]•3H_2_O, polyvinylpyrrolidone (PVP, K30), Calcium chloride anhydrous (CaCl_2_), doxorubicin hydrochloride (DOX), hydrochloric acid (HCl, 36.0–38.0%) were purchased from Chengdu Macklin Biochemical Co., Ltd. Hydrogen peroxide(H_2_O_2_, 30%), Hydrogen Peroxide(H_2_O_2_) Content Assay Kit, glutaraldehyde (25%, GA) were supplied by Solarbio Inc. (Beijing, China). CCK8 and EdU-488 cell proliferation kits, 3,3′,5,5′-Tetramethylbenzidine (TMB), 4′,6-diamidino-2-phenylindole dihydrochloride (DAPI) were bought from Shanghai Titan Technology Co., Ltd. Dulbecco’s modified Eagle medium (DMEM), antibiotics, trypsin–EDTA and phosphate buffered saline (PBS) were obtained from Gibco-BRL (Burlington, ON, Canada). Fetal bovine serum (FBS) were obtained from Inner Mongolia Opcel Biotechnology Co., Ltd. Reactive oxygen species (ROS) fluorescent probe-dihydroethidium (DHE) was purchased from Suzhou Yuheng Biological T echnology Co., Ltd. The AO/EB Double Staining Kit was purchased from Sangon Biotech (Shanghai) Co., Ltd. Ethanol (CH_3_CH_2_OH) (> 99%), ammonia solution (NH_3_•H_2_O, 25 wt%), polyacrylic acid (PAA, M.W. ~ 3000) were purchased from Aladdin, Inc (Shanghai, China). All reagents were used as received without further purification. The deionized water used in all experiments was produced by a Milli-Q water purification system with a specific resistance greater than 18 MΩ cm at 25 °C.

### Characterization

The TEM images were recorded on a JEOL JEM-1200EX (Japanese Electronics and America GATAN). The DLS and Zeta potentials were obtained by NanoBrook Omni particle sizer (Brookhaven Instruments, USA). The XRD pattern was recorded on a D8 ADVANCE diffractometer (Brucker, Germany). The UV–Vis absorption curve was obtained using a UV-2550 Spectrophotometer (Shimadzu, Kyoto, Japan). Fourier transform infrared spectroscopy (FTIR) spectra were collected on a Nicolet IS10 (American Thermoelectric). The XPS were measured using a Thermo Escalab 250Xi (Thermo Scientific Escalab, USA). Specific surface area and corresponding pore-size distribution were determined on a Micromeritics Tristar 3000 system (ASAP, 2460, Micromeritics, USA) and tested by the nitrogen (N_2_) adsorption–desorption isothermal method. CCK-8 content were recorded using a microplate reader. The fluorescence images of Live and Dead, intracellular reactive oxygen species (ROS) and were obtained by an integrated fluorescence microscopic imaging system (Keyence, China). Cell uptake image and EdU-488 cell proliferation assay were observed by confocal laser scanning microscopy (CLSM).

### Preparation of HPB Nanoparticles

HPB was made using a previously reported method with slight modifications^32^. Briefly, K_3_[Fe (CN)_6_] •3H_2_O (132 mg) and PVP (3 g) were dissolved in a 0.01 M HCl solution (40 mL) and sonicated until fully dissolved. The mixture was then incubated at 80 °C for 24 h and cooled, centrifuged, and washed with deionized water and anhydrous ethanol to obtain PB. PB was converted to HPB by dissolving 20 mg PB nanoparticles and 100 mg PVP in a 1.0 M HCl solution (20 mL), stirring for 3 h, heating at 140 °C for 4 h, then cooling, centrifuging, rinsing with ethanol and deionized water, and finally freeze-drying. HPB-PAA was made by stirring HPB with 10 mL of PAA/ethanol solution (5% w/w) for 12 h, centrifuging, washing with deionized water and ethanol, then lyophilizing in the dark.

### Synthesis of CaO_2_ nanoparticles

CaO_2_ nanoparticles were made using a method based on previous literature with a slight modification^22^. The process involved dissolving 1 g of CaCl_2_ in 40 mL ethanol (> 99%), then adding 1 mL of ammonia solution (NH_3_•H_2_O, 25 wt%) under stirring. Next, 0.2 mL of H_2_O_2_ was added dropwise over 4 min with constant stirring, resulting in a light blue solution. The mixture was then centrifuged at 12,000 rpm for 15 min and washed three times with methanol. Finally, the white CaO_2_ product was dried at 50 °C under vacuum for 4 h.

### Synthesis of PCDP

HPB(5 mg), CaO_2_(10 mg) and DOX (10 mg) were added into a glass bottle with ethanol (20 mL) under gentle stirring for 24 h. Then put 10 mL PAA/ethanol solution (5% w/w) into the bottle and stir for another 12 h to obtain PCDP. In addition, HPB@CaO_2_/DOX nanoparticles can be obtained without adding PAA and other steps remain unchanged.

### Drug loading in vitro

HPB (5 mg), CaO_2_ (10 mg), and DOX (10 mg) were mixed with ethanol (20 mL) and stirred for 24 h. PAA/ethanol solution (5% w/w) was then added and stirred for another 12 h. The resulting PCDP was acquired by centrifugation, washed with ethanol, and weighed after lyophilization. The supernatants were collected and analyzed to determine the absorbance of free DOX using UV–Vis spectroscopy at 480 nm^33^. Then HCl solution was added, the quality of CaO_2_ in the supernatant was measured via the volume of supernatants and the concentration of Ca obtained by ICP-MS^34^. Simultaneously, Standard curves of DOX were made by the linear correlation between the absorbance and concentration. The percentage of drug loading was calculated reverse by testing residual drug concentration in the supernatant after drug loading and centrifugation. The loading capacity was calculated based on the following formula:^34^ loading capacity (%, w/w) = ((total DOX/CaO_2_-unloaded DOX/CaO_2_)/total DOX/CaO_2_) × 100%. The loading capacity of HPB@CaO_2_/DOX was evaluated with a similar procedure.

### Drug release in vitro

Precisely weighted HPB@CaO_2_/DOX and PCDP were dispersed in a specific volume of PBS with varying pH levels (pH 7.4 and pH6.5), mimicking the conditions of normal tissues and TME^35^, and swirled at room temperature. The solution was then centrifuged, the concentration of Ca and free DOX in supernatant was estimated using ICP-MS and the characteristic peak at 480 nm in the UV–Vis spectroscopy at specific time intervals over 2 h. Subsequently, the release profiles were derived from a range of obtained concentration of free DOX and CaO_2_ to depict the release process.

### Photothermal performance of PCDP in Vitro

Samples of PBS, HPB, HPB@CaO_2_/DOX, PCDP solutions(0.2 mg/ml) were irradiated with 808 nm NIR laser (1 W/cm^2^, 5 min), and the temperature changes were recorded. meanwhile, UV–Vis spectroscopy of HPB and PCDP was record before and after laser irradiation for 300 s. Under the irradiation of 808 nm laser, different power densities (0.4, 0.6, 0.8, 1.0, 1.2 W/cm^2^) were used to treat 200 ug/ml PCDP solution. With the constant power density (1 W/cm^2^), the photothermal performance of different concentrations (25, 50, 100, 200, 400 ug/ml) of PCDP solutions was recorded. In photothermal stability tests, PCDP solution was irradiated under 808 nm laser for 5 min, thermometer was used to record the real-time temperature of the solution. Then turned the laser off, the solution cooled down to room temperature without other treatment. Four on/off cycles were repeated and measured. To evaluate the photothermal conversion efficiency, the heating–cooling processes of PCDP solution and water were recorded respectively, then the results are obtained by fitting and calculation^36–38^.

### H_2_O_2_ generation

In short, weighted PCDP were scattered in PBS with different pH (pH7.4 and pH6.5), and incubated at room temperature for 0–4 h. Afterward, the H_2_O_2_ levels were quantified using the Hydrogen Peroxide (H_2_O_2_) Content Assay Kit (Solarbio) at intervals of 1 h^39^.

### Chemodynamic activity

3,3′,5,5′-Tetramethylbenzidine (TMB) can be oxidized to the oxidation state of TMB by •OH generated by PCDP under acidic environment, which exhibits a characteristic UV–vis absorbance at 655 nm. Briefly, different densities of PCDP and TMB(8 mM, 300 ul) were mixed in PBS with different pH values. The UV–vis spectra of various samples were detected by UV–vis spectrophotometer^39^.

### Hemolysis assay

Hemolysis test^40^ was carried out using fresh anticoagulant blood collected from the First People’s Hospital of Chongqing Medical University. Red blood cells (RBCs) were separated at 3000 rpm for 5 min, washed multiple times with sterile isotonic PBS until the supernatant was clear, and then diluted tenfold with PBS. Afterwards, 100 μL diluted RBCs suspension were combined with 900 μL of PCDP (concentrations of 10, 5, 2.5 and 1.25 mg/mL), maintained for 2 h at 37 °C. Meanwhile, 100μL of RBC solution dissolved in clean water and PBS were used as positive and negative controls, respectively. The absorptions of supernatant were detected at 540 nm by UV–vis spectrophotometer after 2-h culture.

### Cytotoxicity evaluation

The toxic effects of nanoparticles on CRC cells were assessed using CCK-8 assay. Briefly, Caco-2 cells were cultured into 96-well plates, at logarithmic growth phase (1 × 10^4^). Once the cells adhered, different concentrations of HPB, HPB-PAA, CaO_2_ and PCDP were added to well and cultured for 24 h, though PBS was served as the control. Subsequently, the medium was changed with 100 μL fresh ones including 10 μL CCK-8 solution. The optical density (OD) of each well was measured at 450 nm using a spectrophotometric plate reader after 2-h culture. Survival rate of the cell control group was set to 100%. These tests were carried out three times in duplicate.

### Cellular uptake

Caco-2 cells were grown on cell climbing slices in 12-well plates. Once the cells adhered, the medium was changed with new one including 10 µg/mL PCDP, proceeded by incubation for 0, 1, 2, and 4 h. The cells were cleaned with PBS, fixed in 4% paraformaldehyde, then stained with DAPI. The contents were exposed to CLSM, with red and blue fluorescence signifying incorporated nanoparticles and nuclei, respectively. Furthermore, cell uptake of PCDP was also evaluated using bio-TEM. Briefly, cells incubated with PCDP for 4 h were prepared after digesting, centrifugating, washing and resuspending overnight in glutaraldehyde. Afterward, cells were fixed before being sent to Beijing Zhongkebaice Technology Co., LTD for Bio-TEM examination.

### Cell viability assays

Caco-2 cells were cultured in 6-well plates until reaching about 80% confluence. The cells were treated with different experimental groups including HPB, CaO_2_, DOX, PCDP, and PCDP + NIR, while samples treated with PBS were utilized as controls. The cells of PCDP + NIR group was additionally irradiated with an 808 nm laser for 5 min after incubating PCDP and cells together for 12 h. After incubating for 24 h, the cells were stained with acridine orange/ethidium bromide (AO/EB) for 5 min and observed under an integrated fluorescence microscopic imaging system (Keyence, China). Green and red fluorescence indicated live and dead cells, respectively.

### Cell proliferation assay

The Ed Utes was utilized to analyze the cell proliferation capacity. In brief, Caco-2 cells were cultured onto cell climbing slices in 12-well plates until reaching about 70% confluence. Then the medium was replaced with fresh ones including PBS, HPB, CaO_2_, DOX, PCDP and PCDP + NIR incubated for 24 h. similarly, the PCDP + NIR group was irradiated with 808 nm laser for 5 min when the PCDP and cells were incubated for 12 h together. Finally, according to the kit instructions, the fluorescence intensity was observed by confocal laser scanning microscopy (CLSM).

### Intracellular ROS assays

The Caco-2 cells were coped with different groups for 24 h at 37 °C. Next, the medium was discarded and the cells were treated with 1 mL of a solution containing the ROS probe [DHE (1 μM)] and incubated for 30 min in the dark at 37 °C. The supernatant was removed, and the activated DHE fluorescence was captured using an integrated fluorescence microscope imaging system (Keyence, China).

### Determination of cell apoptosis

To investigate cell apoptosis, Caco-2 cells were cultured in 12-well plates until reaching about 70% confluence. Fresh medium including PBS, HPB, CaO_2_, DOX, PCDP, and PCDP + NIR was added and incubated for 24 h. Treated cells were collected with trypsin, rinsed twice with PBS, and stained using the Annexin V/PI cell assay kit, following the instructions provided by the manufacturer. Finally, the samples were analyzed using flow cytometry (FCM) to determine the apoptosis rate.

### In vivo study

Female Balb/c-nu mice aged 5 weeks were acquired from Beijing HFK Bio-Technology.co., LTD. All experimental procedures were authorized by The First Affiliated Hospital of Chongqing Medical University Chongqing and carried out strictly in accordance with protocol guidelines. 150 μL PBS with 1 × 10^6^ HCT116 cells were injected into the right side back of each mouse to establish the tumor-bearing mouse model. The mice were casually assigned into six groups for in vivo tests (3 mice per group) when the tumor volumes reached approximately 80 mm^3^ (planed as day 0) and intravenously injected with different therapies: PBS (10 ul/g) + NIR group, HPB (10 mg/kg) + NIR group, CaO_2_ (2.1 mg/kg) + NIR group, DOX (5 mg/kg) + NIR group, PCDP (16.61 mg/kg) group, PCDP (16.61 mg/kg) + NIR group. After 24 h injection, tumor location was treated twice with 808 nm NIR (1 W/cm^2^) for 5 min at intervals of 10 min. The tumor size and body weight of mice were measured every 2 days for 14 days. The tumor volume (V) was calculated as: V = (L × W^2^)/2, and the relative tumor volume (V/V_0_) was normalized to the initial volume (V_0_). After receiving therapy for 2 weeks straight, the mice were killed by cervical dislocation, and solid tumors were excised and imaged. To assess histological changes and in vivo PCDP biocompatibility. The tumors and major organs were sliced and stained with hematoxylin and eosin.

### Ethics approval and consent to participate

The study is reported in accordance with ARRIVE guidelines and animal studies in the present study were approved by the Ethics Committee of Animal Experiments of The First Affiliated Hospital of Chongqing Medical University Chongqing. The CRC cell lines HCT116 and Caco-2 were purchased from the Chinese Academy of Sciences Cell Bank (Shanghai) and stored in liquid nitrogen. Euthanasia method of nude mice: Cervical dislocation. Procedure: The operator holds the tail of the nude mouse with their right hand, places the mouse on the experimental table, holds the head and neck of the mouse with their left hand, and exerts force to pull the tail backward and upward with the right hand. When the operator feels the spine of the animal disconnect, the animal dies immediately.

## Results and discussion

### Synthesis and characterization of PCDP

**Figure 2:**
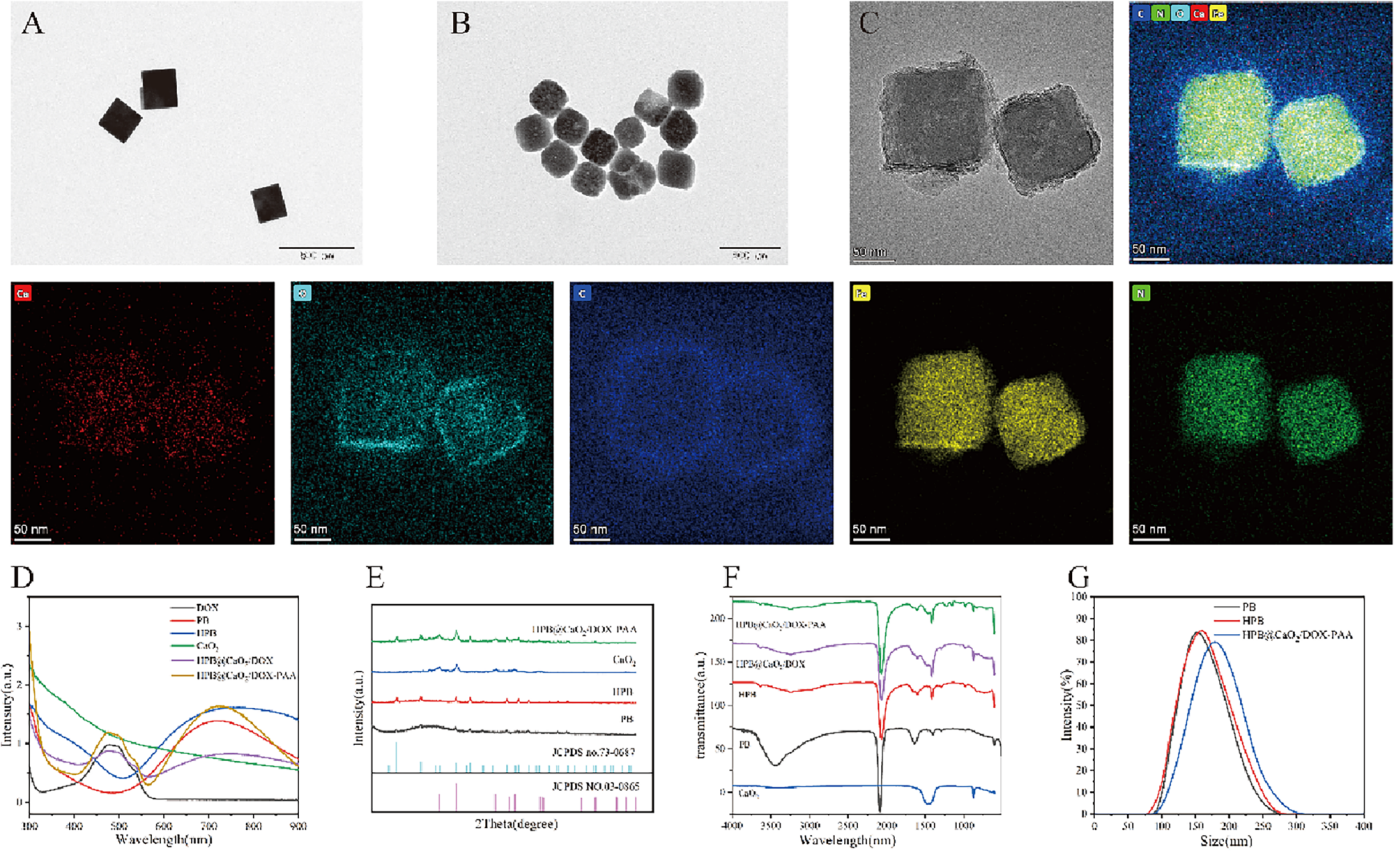
(**A**) TEM image of PB. (**B**) TEM image of HPB. (**C**) TEM image of PCDP and EDS-mapping of PCDP. (**D**) UV–vis spectra. (**E**) XRD pattern. (**F**) FTIR spectra. (**G**) Dynamic light scattering (DLS) particle size distributions (concentration: 0.1 mg/ml).

The preparation procedure for TME-activated PCDP is shown in Fig. 1. Following the initial synthesis of PB and CaO_2_, hollow mesoporous HPB was created by etching PB with hydrochloric acid. HPB were cleaned and purified before being loaded with CaO_2_ and DOX and having PAA applied to increase their biocompatibility. HPB@CaO_2_/DOX control groups were concurrently created. The diameter of PB, HPB, and PCDP was approximately 109 nm, 110 nm, and 140 nm, respectively, according to the TEM picture in Figs. 2A–C. According to DLS measurements, the typical hydrated diameters of PB, HPB, and PCDP were around 152.5 nm, 159 nm, and 178.1 nm, respectively (Fig. 2G). Due to the related hydration layer, the diameters measured by DLS were greater than the TEM measurements. By EPR effects, the right nanoparticle sizes facilitate aggregation at the tumor location and longer blood circulation times. Moreover, results from element map scanning demonstrated that PCDP included Fe and Ca, two elements that are typical of HPB and CaO_2_ (Fig. 2C).

The specific surface area and porous structure of HPB were next examined using a nitrogen adsorption–desorption experiment. HPB displayed a classic Langmuir type IV isotherm, as seen in Fig. S1B, which denotes the existence of mesopores. It had a surface area of 244.8948 m^2^/g and pore sizes of 3.56 nm^41^. Zeta potential analysis was used to assess the potential changes before and after CaO_2_ and DOX loading and surface alteration. When dispersed in deionized water, HPB had a negative potential of -17.76 mV, as illustrated in Fig. S1A. After CaO_2_ and DOX loading, the zeta potential was measured to be at − 3.73 mV, and it further dropped to − 16.54 mV following further PAA coating.

The synthetic architectures of nanoparticles were examined using UV–Vis absorption spectroscopy. According to the findings, the absorption band for PB, HPB, HPB@CaO_2_/DOX, and PCDP was wide and ranged from 600 to 800 nm (Fig. 2D)^41^. The characteristic peak of DOX at 480 nm in the UV–Vis spectrum revealed that DOX was enclosed in the nano-catalyst.

The successful CaO_2_/DOX loading and PAA coating during the production of CaO_2_@HMSNs-PAA were further confirmed using the FT-IR spectra. As seen in Fig. 2F, the stretching vibration of the PVP amide group and the C–N bond vibration in PB cause distinctive peaks to appear between 2070 and 1605 cm^−1^^42^. The characteristic peaks near 1414 cm^−1^ and 875 cm^−1^ result from C–C and O–O vibrations in DOX and CaO_2_, respectively^43^, and the new absorption peaks of PCDP at 1226 cm^−1^ and 1155 cm^−1^ were produced by stretching vibrations of amide I and amide II, respectively^44^, indicating that chemical cross-linking has been formed between HPB and its coated PAA.

In addition, we also examined the nanoparticles' X-ray diffraction (XRD) spectra. According to Fig. 2E, HPB and CaO_2_ were identified in the chemical structure by the XRD pattern, which was consistent with the standard card (JCPDS cards 73-0687 and 03-0865)^22,41^. The diffraction peak of HPB and CaO_2_ could be seen in the XRD pattern of the PCDP sample, showing the coexistence of the two phases in the sample.

The effective synthesis of PCDP was also validated by X-ray photoelectron spectroscopy (XPS). As can be seen in Figure S2A–D, Fe 2p_3/2_ and Fe 2p_1/2_, which are the properties of Fe^3+^ generated from Fe_4_[Fe (CN)_6_]_3_, are responsible for the peaks at 711.1 and 724.8 eV, whereas Fe 2p_3/2_ from [Fe (CN) _6_]^4−^ is responsible for the peak at 708.2 eV. Ca 2p_3/2_ and Ca 2p_1/2_ have peaks at 346.6 and 350.2 eV, respectively. O1s, which are produced from the O–O bond of CaO_2_, account for the 531.5 eV peak^45,46^.

Finally, the diameters of the nanoparticles measured by DLS were 190, 191.7, and 194.4 nm, respectively after 3 days at room temperature when using deionized water, PBS buffer (pH 7.4), and cell culture medium (containing serum) as dispersed phases (Fig. S3A–C), indicating the stability of PCDP. These characterization data collectively demonstrated that the PCDP was successfully synthesized.

### Drug encapsulation and release in vitro

During the production of PCDP and HPB@CaO_2_/DOX, CaO_2_ and DOX penetrate the mesoporous channels and inner cavities of the prepared HPB through simple molecular diffusion in the CaO_2_/DOX/ethanol solution. To quantify the drug loading, a standard curve of DOX concentration versus absorbance was first made (Fig. S4A, B). Then, the loading content of CaO_2_ and DOX in PCDP and HPB@CaO_2_/DOX could be calculated based on the absorbance of free DOX measured by UV–Vis absorption spectroscopy in the supernatant and the concentration of Ca^2+^ determined by inductively coupled plasma (ICP) element analysis in the supernatant after encapsulation, respectively. Owing to the small leakage of CaO_2_/DOX and the addition of PAA during PAA coating, the loading capacity of CaO_2_/DOX in PCDP and (24.58%, 30.1%) was somewhat lower than that of HPB@CaO_2_/DOX (25.16%, 42%).

**Figure 3:**
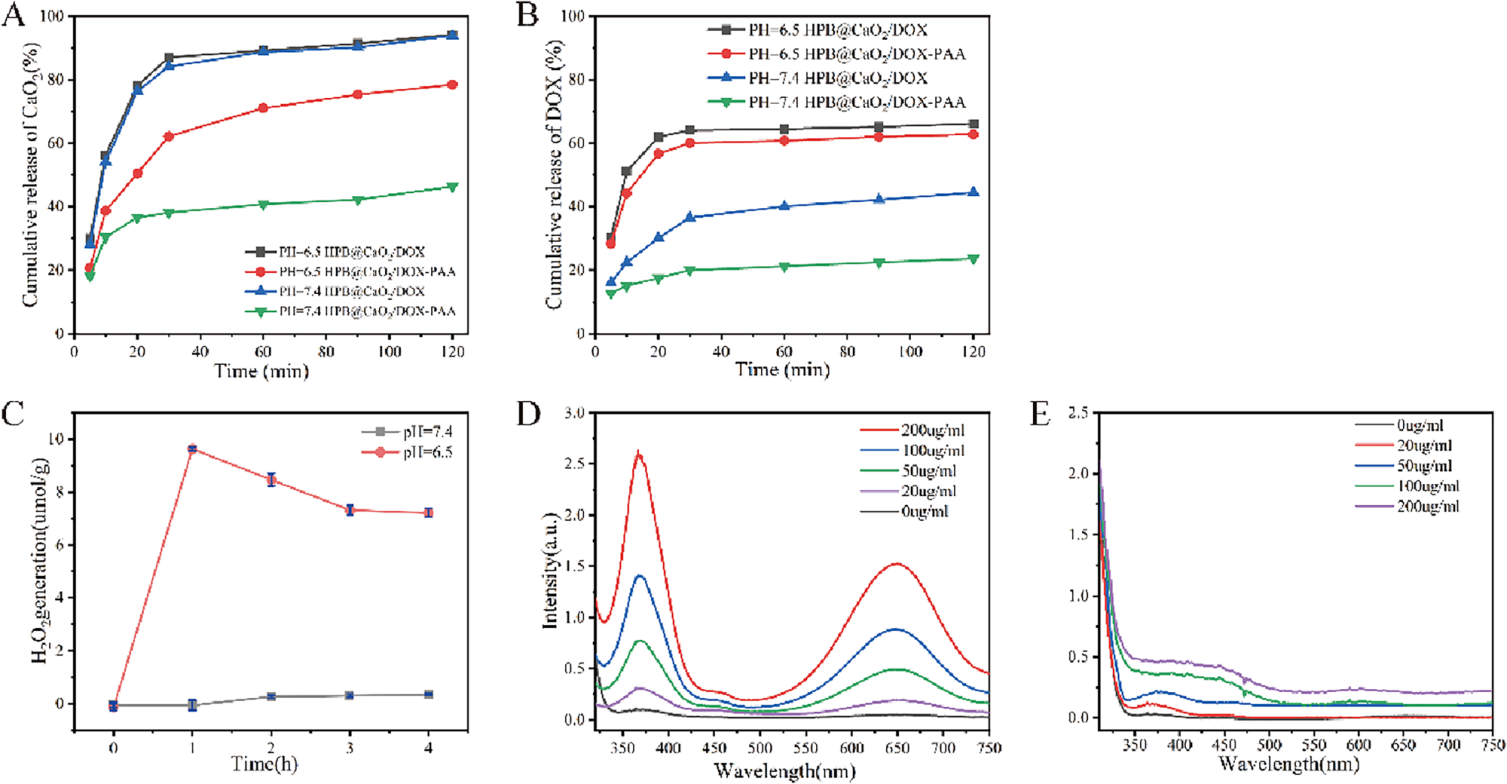
(**A**) CaO_2_ and (**B**) DOX release profiles from PCDP and HPB@CaO_2_/DOX in buffer solutions with different pH values. (**C**) H_2_O_2_ release profile of the PCDP at different pH values. UV–vis spectra of the PCDP with varied concentrations in TMB solutions of (**D**) pH = 7.4 and (**E**) pH = 6.5.

The quantities of Ca^2+^ in suspensions identified by ICP-MS and the absorbance of DOX in suspensions obtained by UV–Vis absorption spectroscopy were similarly used to evaluate the CaO_2_ and DOX release patterns. Figure 3A,B depicts the CaO_2_ and DOX release patterns from PCDP and HPB@CaO_2_/DOX in PBS (without calcium and magnesium) at pH 7.4 and 6.5, respectively. These PBS were chosen to replicate the environment of normal tissues/blood (pH 7.4) and TME (pH 6.5). The release of CaO_2_ and DOX from the two nano-catalysts was constant in solutions with two different pH values, although the release rates varied. The release patterns of HPB@CaO_2_/DOX in PBS buffers with different pH values for CaO_2_ and DOX were found to be very similar. At 30 min, the release rates of CaO_2_ were 84.12% at pH 7.4 and 87.02% at pH 6.5. The release rates of DOX were 36.58% at pH 7.4 and 64.07% at pH 6.5. At 120 min, the release rates of CaO_2_ reached 93.82% at pH 7.4 and 94.17% at pH 6.5. The release rates of DOX were 44.46% at pH 7.4 and 66.17% at pH 6.5. While, owing to the presence of PAA coating, the CaO_2_ and DOX release mode of PCDP was considerably different from that of HPB@CaO_2_/DOX and altered with the pH of PBS. Its pH-responsive release was created as a result of PAA's distinctive swelling and flexibility behavior under various pH-valued situations. The coated PAA layer could effectively cover the pores in HPB and prevent the CaO_2_ and DOX from leaking when the pH of PBS was raised to 7.4, which could also enhance the stability of CaO_2_ and DOX. The swelling ratio of PAA noticeably increased after exposure to the simulated acidic TME (pH 6.5), partially liberating the pore outlets. Moreover, the osmotic pressure insured that more H_2_O would permeate into the mesoporous channels of HPB from PBS, increasing the release of CaO_2_ and DOX. While CaO_2_ and DOX release rates from PCDP may reach as high as 62.14% and 60.08% at pH 6.5, they were only 38.11% and 20.01% at 30 min at pH 7.4, respectively. While the release rates of CaO_2_ and DOX from PCDP could reach as high as 78.44% and 62.77% at pH 6.5, respectively. They gradually climbed to 46.31% and 23.68% at pH 7.4 when the exposure duration was prolonged to 120 min. Based on the aforementioned findings, PCDP might transport more CaO_2_ and DOX to the tumor site by releasing less CaO_2_ and DOX in normal tissues and reducing the needless loss of CaO_2_ and DOX during blood circulation. The responsive release of medications in vivo is nonetheless sufficiently supported by this in vitro release experiment, even if it is challenging to perfectly imitate the real in vivo release mechanism.

### H_2_O_2_ generation and chemodynamic activity

Figure 3c illustrated that different pH levels were employed to evaluate the generation of H_2_O_2_ by the nano-catalysts. When the solution was in a neutral state, there was no evident evidence of H_2_O_2_ production from PCDP. However, upon altering the pH level of the solution to 6.5, H_2_O_2_ production could be rapidly induced. It is interesting to observe that after achieving the highest magnitude, the amount of H_2_O_2_ created drops. This phenomenon could potentially be attributed to the dissociation of PCDP under acidic conditions, which triggers rapid H_2_O_2_ formation. Subsequently, the produced H_2_O_2_ gets consumed by Fe^2+^ to generate hydroxyl radicals (•OH), leading to the decrease in H_2_O_2_ level. Therefore, we verified the presence of •OH generation, as TMB has a characteristic absorbance at approximately 650 nm due to the oxidation of TMB by •OH. We dissolved appropriate amount of PCDP with PBS of different pH values, then mixed with TMB solution, finally observed the UV–visible spectrum of the mixture. As shown in Fig. 3D, the characteristic absorption peak at 655 nm (ox-TMB) could be observed in the UV spectrum at pH = 6.5, and there was an obvious color change of the mixture. Meanwhile, the rate of color development reaction was accelerated with the increase of PCDP concentration, showing a typical concentration dependence. On the contrary, at pH = 7.4, there was no obvious absorption at the same characteristic peak position (Fig. 3E). The above results indicated that the Fenton reaction of PCDP occurs under acidic conditions.

### Photothermal performance of PCDP

**Figure 4:**
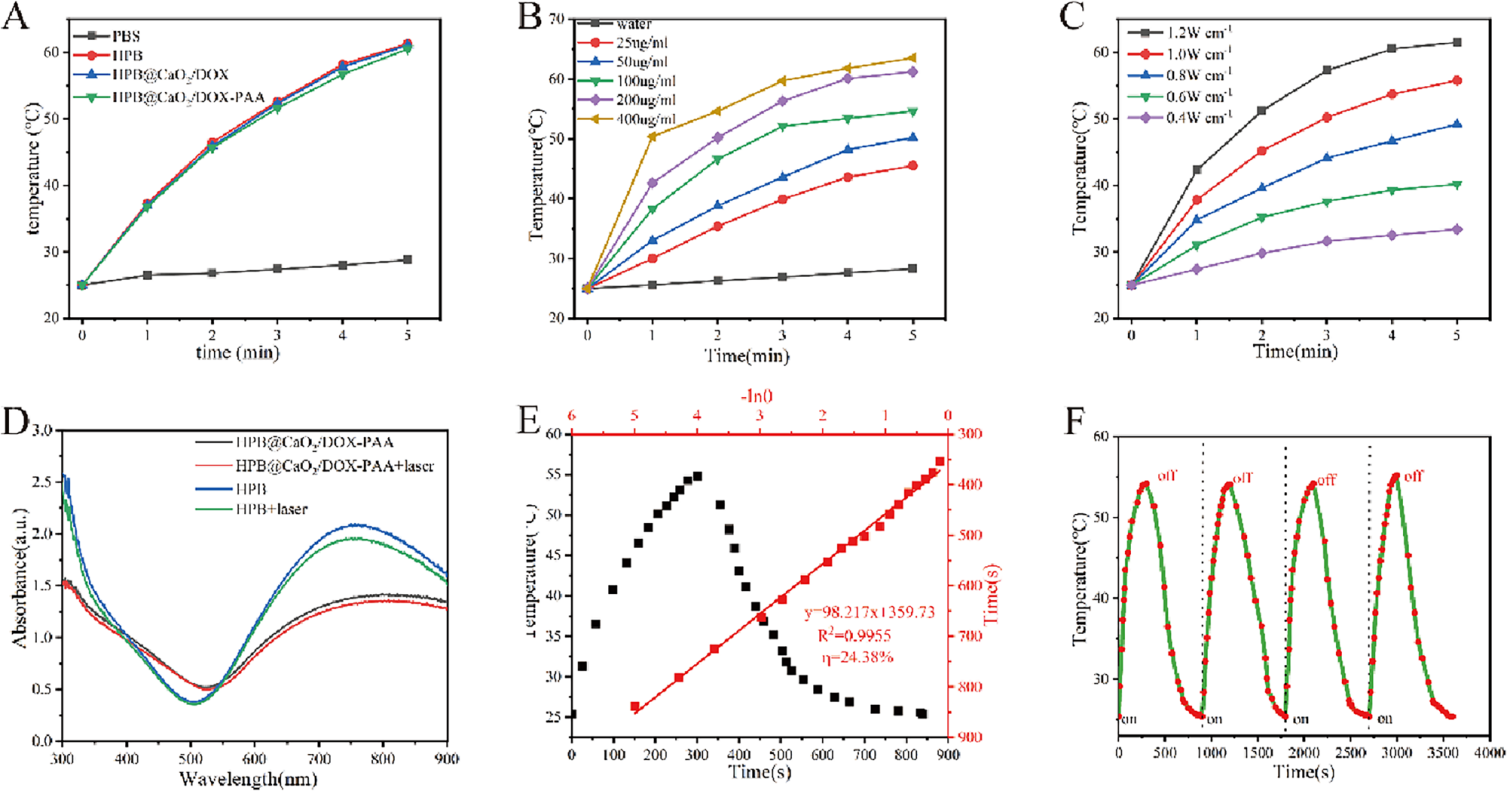
(**A**) Temperature change of different samples (0.2 mg/ml) after with 808 nm laser irradiation (1 W/cm^2^) for 300 s. (**B**) Photothermal performance of the PCDP (0.2 mg/ml) under different laser power (0.4, 0.6, 0.8, 1.0, 1.2 W/cm^2^). (**C**) Photothermal performance of the PCDP with different concentrations (25, 50, 100, 200, 400 ug/ml.laser:1 W/cm^2^). (**D**) UV–vis spectra of HPB and PCDP before and after with 808 nm laser irradiation. (**E**) Photothermal curve of PCDP aqueous dispersion (1 ml, 0.2 mg/ml) during on and off laser (1 W/cm^2^). Linear cooling time versus—Ln(θ) of the PCDP acquired from photothermal curve. (**F**) Photothermal stability of the PCDP under 808 nm laser irradiation. (heating/cooling time: 9 min, PCDP: 0.2 mg/ml, laser:1 W/cm^2^).

The study investigated the photophysical properties of PCDP in aqueous solutions. When exposed to an 808 nm laser (1 W/cm^2^) for 5 min, the temperature of HPB, HPB@CaO_2_/DOX, and PCDP quickly increased to above 50 °C, which is the temperature that can efficiently destroy tumors (42.6 °C). Furthermore, the temperature trend was consistent among all three groups (HPB, PCD, and PCDP). However, the temperature of PBS only increased to 28.8 °C (Fig. 4A). According to the results, HPB, HPB@CaO_2_/DOX, and PCDP all demonstrated similarly favorable photothermal effects in vitro. The drug loading and PAA wrapping did not significantly affect their photothermal properties. Next, Fig. 4B,C showed that as the laser power and PCDP concentration increased, the temperature of the PCDP solution also increased, indicating that the photothermal effect was directly proportional to both of these factors. Furthermore, the UV–vis spectra of HPB and PCDP before and after laser irradiation depicted in Fig. 4D demonstrated that both materials possessed exceptional photothermal stability, as their UV absorption peaks remained largely unchanged. Simultaneously, we monitored the temperature of PCDP during all four heating and cooling cycles, as depicted in Fig. 4E,F. Our observations indicated that there was no noticeable discrepancy in the maximum temperature reached by PCDP during each cycle, leading us to determine that the photothermal conversion efficiency (η) of PCDP was 24.38%. In comparison to gold nanoparticles, PCDP exhibited a higher photothermal conversion efficiency of 22%^47^. Collectively, these findings demonstrate that PCDP boasts exceptional photostability and superior photothermal conversion efficiency, positioning it as an excellent photothermal nanomaterial.

### Hemolysis assay

As depicted in Fig. 6F, the results obtained from the standard hemolysis assay conducted to evaluate the hemocompatibility of PCDP samples revealed that no apparent hemolysis effect was observed in comparison to visibly red positive controls for any concentration of PCDP samples. Even at a PCDP concentration of 10 mg/ml, the hemolysis rate was only 3.5%. These findings were also validated by the UV–vis absorption spectroscopy results of the supernatant. The favorable biocompatibility of the nano-catalysts with blood cells was confirmed by the low hemolytic activity of PCDP, which is advantageous for intravenous administration.

### Intracellular uptake

**Figure 5:**
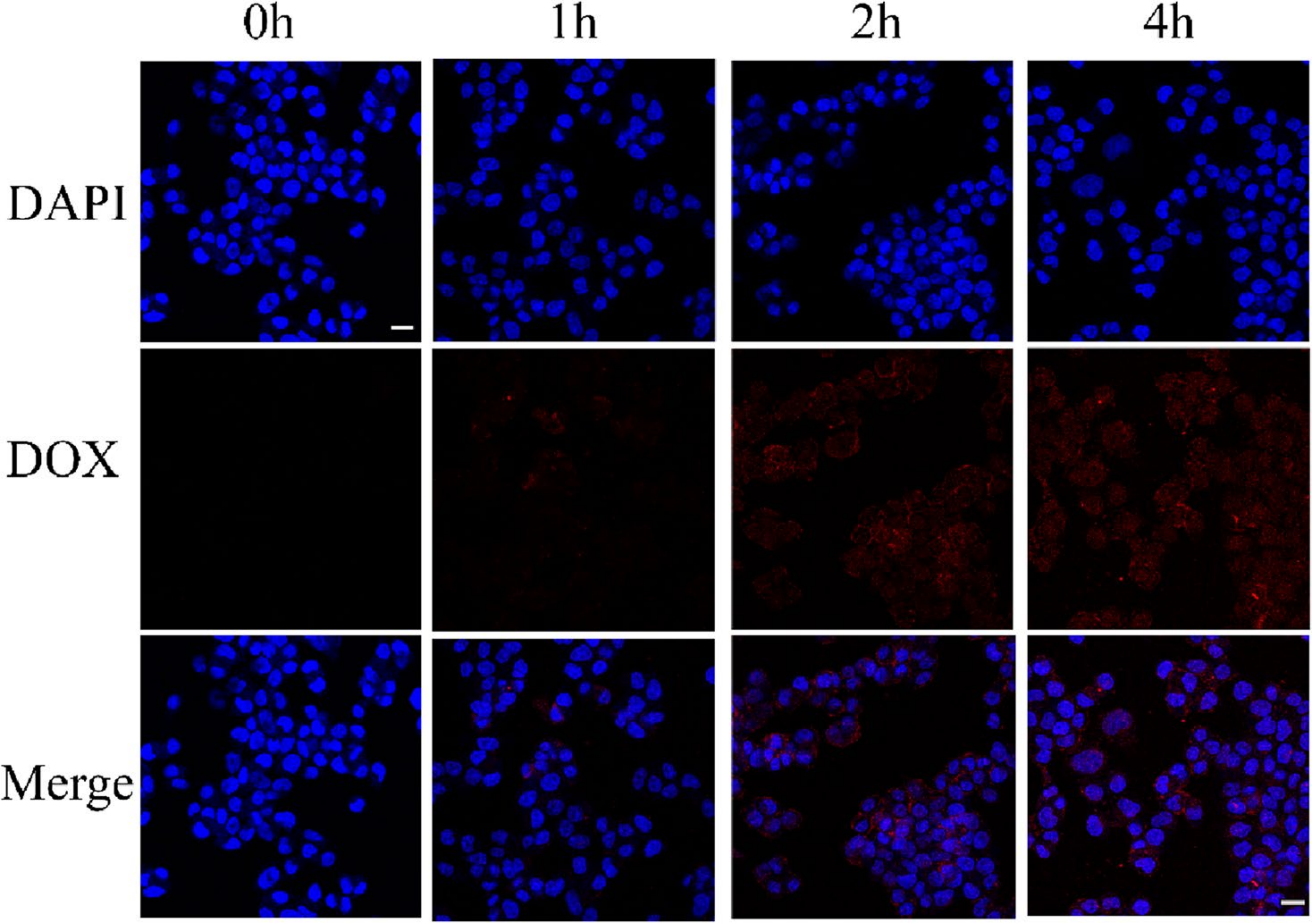
Intracellular uptake of PCDP in Caco-2 cells. Confocal laser scanning microscopy images of Caco-2 cells describing the intracellular uptake of PCDP containing DOX (red) with the nuclei stained by DAPI (blue) after incubation for 0–4 h. Scale bar, 50 μm.

According to literature findings, endocytosis is the main cellular uptake mechanism for nanomaterials that have a diameter smaller than 500 nm^48^. It was hypothesized that the entry of PCDP into cells followed the endocytosis pathway, as the PCDP produced in this study had a diameter of approximately 178.1 nm. The confirmation of this finding was observed using a laser confocal scanning microscope (CLSM) to visualize the endocytosis of the materials (Fig. 5). PCDP nanoparticles were tracked through DOX's red fluorescence, while the nucleus was labeled using DAPI dye. The results indicated that the fluorescence increased with the incubation duration, suggesting that the cells could efficiently internalize PCDP into the cytoplasm. Furthermore, Caco-2 cells were co-cultured with PCDP for 4 h and then imaged using bioelectron microscopy to demonstrate the ability of PCDP to penetrate cells. The results revealed the presence of PCDP particles in cytoplasmic vesicles (Figure S5).

### Tumor cell-killing ability in vitro

**Figure 6:**
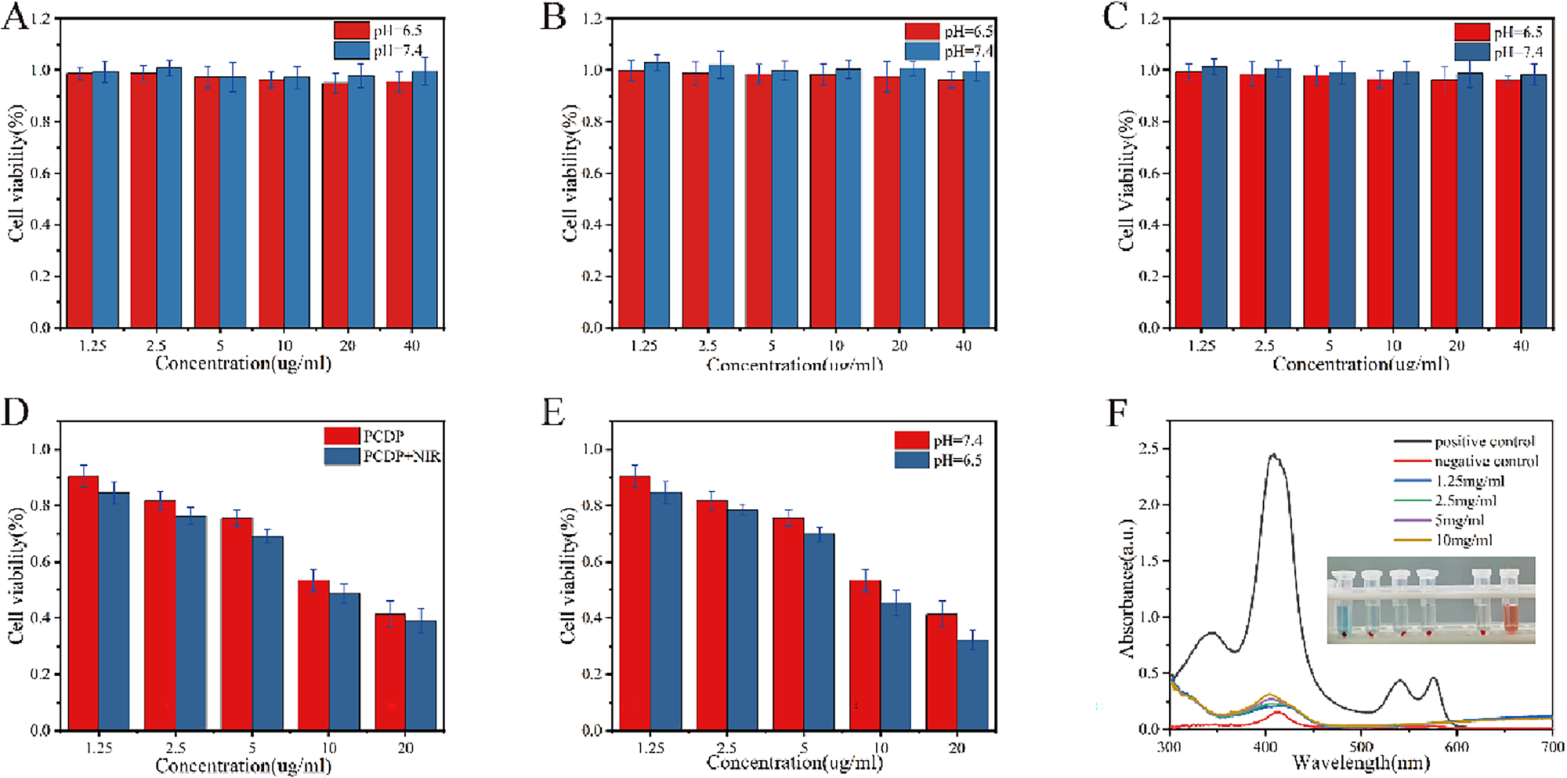
In vitro cytotoxicity of nanomaterial. Relative viabilities of Caco-2 cells after incubation with indicated concentrations of the HPB (**A**) and HPB-PAA (**B**) and CaO_2_ (**C**) for 24 h. (**E**) Relative viabilities of Caco-2 cells after incubation with indicated concentrations of the PCDP for 24 h with pH7.4 and pH 6.0 (**D**), with/without laser irradiation (1 W/cm^2^). (**F**) Hemolysis assay after incubation with different concentrations of PCDP. (UV–vis absorbance spectra of supernatant and Photographs: PC positive control, NC negative control, 10 mg/ml, 5 mg/ml, 2.5 mg/ml, 1.25 mg/ml).

In the current study, the tumor cell-killing ability of PCDP was evaluated in vitro using Cell counting kit-8 (CCK-8) assays, the EdU cell proliferation test, and live/dead (AO/EB) labeling. According to Fig. 6A–E, in the CCK-8 test, the Caco-2 cell activities did not change significantly after co-incubation with HPB, HPB-PAA, and CaO_2_ nanoparticles (at concentrations of 1.25, 2.5, 5, 10, 20, and 40 μg/mL) in pH 7.4 and 6.5 media for 24 h. These results suggested that the three nanoparticles exhibited low toxicity to tumor cells. However, co-incubation of Caco-2 cells with PCDP nanoparticles (at concentrations of 1.25, 2.5, 5, 10, and 20 μg/mL) for 24 h under pH 7.4 and 6.5 conditions significantly reduced cellular activity and exhibited some pH responsiveness. Additionally, the photothermal effect generated by 808 nm laser irradiation also caused enhanced toxicity of PCDP at the same concentration, resulting in a further decrease in cellular activity of Caco-2 cells. These findings suggested that HPB or CaO_2_ nanoparticles alone did not have a significant cell-killing effect, while the PCDP nano-catalysts constructed (including CaO_2_-mediated CDT, HPB-mediated photothermal treatment, and DOX-triggered chemotherapy) are capable of produce more powerful toxic effects on cells.

To differentiate between live and dead cells, the Calcein Acetoxymethyl Ester AO/EB staining assay was conducted. And the results were consistent with those of the CCK8 assay, as depicted in Figure S7. Strong green fluorescence signals were observed in the control (PBS) and HPB groups, indicating that the treatments did not affect the cancer cells. The CaO_2_ group showed minimal red fluorescence and no significant change in the number of cells. In the DOX group, a slight decrease in cell count was observed, along with partial red fluorescence. Furthermore, the group treated with PCDP and PCDP + NIR exhibited intense red fluorescence and a significant decrease in the number of tumor cells. These results further suggested that combination therapy can lead to extensive Caco-2 cell death, while monotherapy has limited effects on cancer cell death.

**Figure 7:**
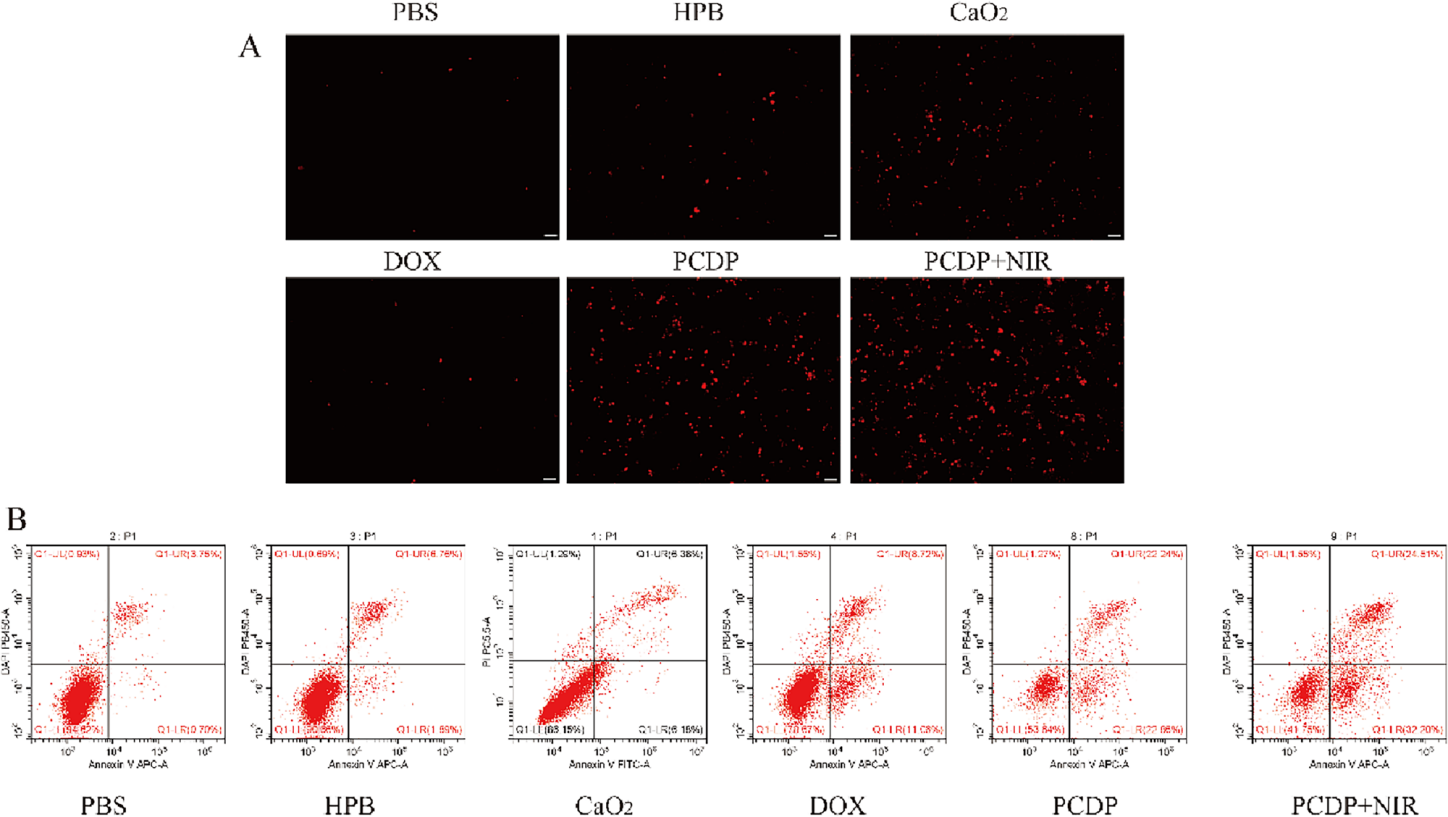
(**A**) Generation of ROS after being treated with PBS, HPB, CaO_2_, DOX, PCDP and PCDP + NIR. Scale bar: 50 μm. (**B**) Apoptosis of Caco-2 cells induced by PBS, HPB, CaO_2_, DOX, PCDP and PCDP + NIR under the conditions of pH 7.4.

An EdU cell proliferation assay (Fig. 7) was conducted to investigate the impact of PCDP on cell proliferation. Results showed that HPB and CaO_2_ treatments had similar effects on cell proliferation as the PBS group, and DOX treatment had very limited effect. On the other hand, PCDP treatment led to a significant inhibition of cell proliferation, particularly in the PCDP + NIR group, where the inhibition was even more pronounced after NIR irradiation.

**Figure 8:**
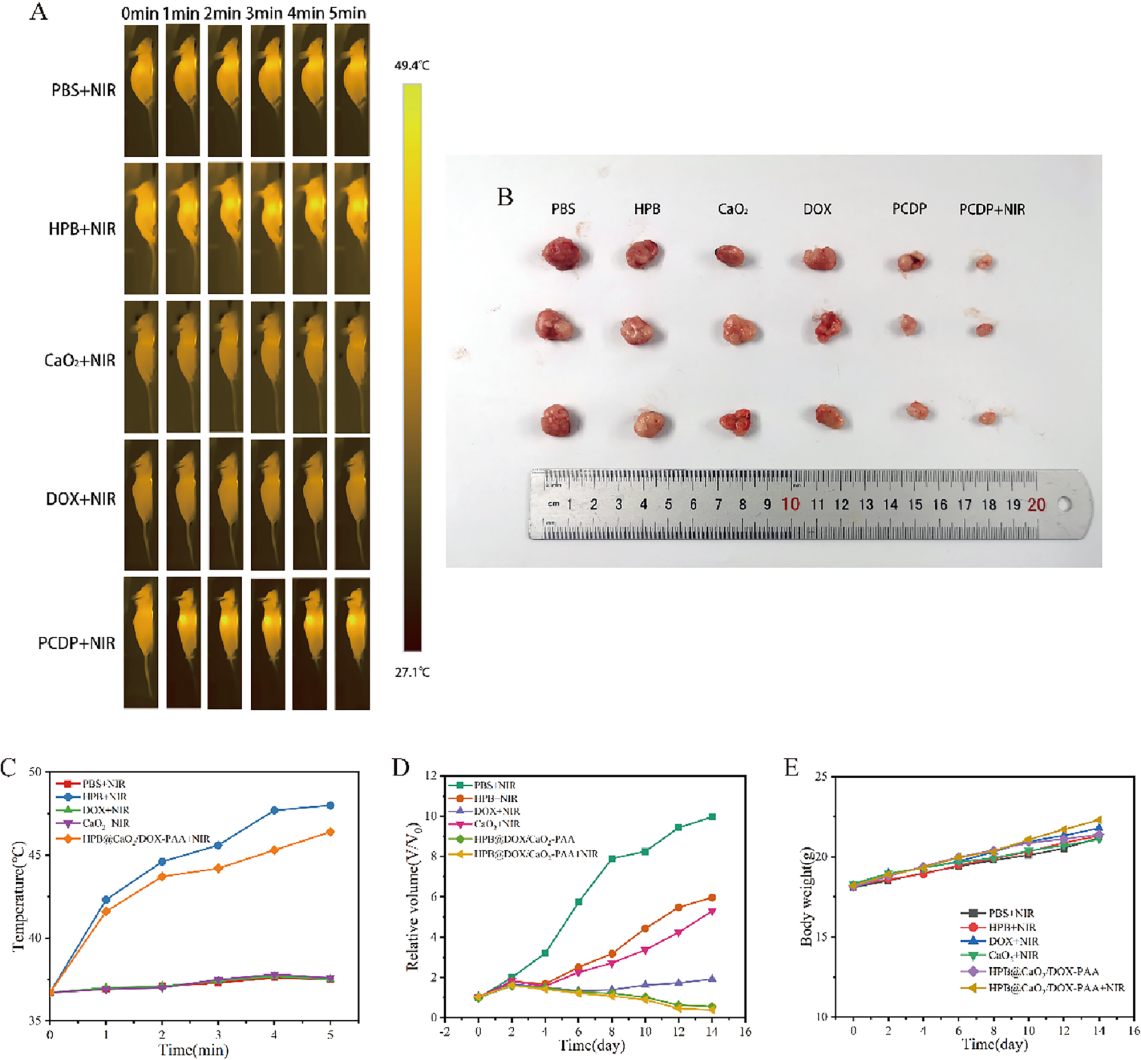
(**A**) IR thermographic images of HCT116 tumor-bearing mice in different groups taken under the exposure to an 808 nm laser (1 W/cm^2^) at different time points. (**B**) Photographs of the final tumor size after 14 days of various treatments. (**C**) The temperature change of the corresponding tumor site recorded in A. (**D**) Relative tumor volume in different treatment groups. (**E**) The change of mice body weight during the treatment period.

### Intracellular ROS production

The ROS probe DHE can effortlessly penetrate and infiltrate the live cell membrane, and oxidized by the intracellular ROS to generate red luminous ethidium oxide, which reflects the overall intracellular ROS level. As a result, we employed a combined fluorescence microscopic imaging system to detect red fluorescence in cells from various treatment groups (Fig. 7A). Only very weak red fluorescence could be seen in the cells of the HPB and CaO_2_ groups, and extra noticeable fluorescence could scarcely be seen in the cells of the DOX group, compared to the minimal red fluorescence caused by auto-oxidation in the negative control group. In contrast, the red fluorescence in the PCDP and PCDP + NIR groups dramatically increased, with the greatest red fluorescence occurring in the PCDP + NIR group's cells following NIR light irradiation. Consequently, it is reasonable to assume that CaO_2_ and HPB are intimately connected to ROS generated in cells and work in concert with one another to produce ROS. Meanwhile all of the aforementioned findings were in line with assessments of in vitro cytotoxicity, it was conceivable that the high levels of intracellular ROS that tumor cells produced and were unable to be effectively eliminated by their overworked antioxidant system would cause severe oxidative stress and ultimately lead to cytotoxicity through the induction of apoptosis.

### Inducing the apoptosis of cancer cells

It was decided to use FCM based on Annexin-V-FITC/PI staining to assess the cell apoptosis caused by PCDP. Those located in the lower right quadrants were regarded as apoptotic cells^49^. According to Fig. 7B, no obvious apoptosis was observed in the HPB group (compared with the negative control group), while the apoptotic rates in DOX and CaO_2_ groups were only 11.06% and 6.18%, and the PCDP and PCDP + NIR groups were higher, 22.86% and 32.20%, respectively. The above results indicated that prepared PCDP nano-catalyst can induce apoptosis, and this effect is more significant after laser irradiation.

### Anti-tumor efficacies of triple therapy in vivo

PCDP had impressive therapeutic benefits in vitro. Thus, the anti-cancer effectiveness of nano-catalysts was assessed in mice. The subcutaneous HCT116 xenografted tumor model was firstly constructed before intravenous injection of several experimental materials. Then after the tumors had grown to a size of around 80 mm^3^, BALB/c nude mice carrying the HCT116 tumor were randomly divided into six groups: the PBS + NIR group (control), the HPB + NIR group, the CaO_2_ + NIR group, the DOX + NIR group, the PCDP group, and the PCDP + NIR group. Treatments are given every 3 days for a total of 14 days. To evaluate the photothermal impact of PCDP in vivo, temperature changes at the tumor site in nude mice were recorded using an infrared thermography (Fotric 286). According to Figs. 8A,C, the tumor temperature in the treatment group containing the HPB component increased rapidly with the duration of NIR laser radiation. After 5 min of NIR laser irradiation, the maximum temperature reached about 46.4 °C, which could achieve effective photothermal treatment. In contrast, the PBS + NIR group only increased by 0.8 °C. This effect was attributed to the strong absorption of NIR light by the HPB nano-catalyst and the low bioinvasiveness of NIR light.

As for tumor treatment results, we photographed tumor specimens harvested from each treatment group at the end of treatment, and the findings revealed that the smallest tumors were found in the PCDP + NIR group (Fig. 8B). We also counted the relative changes of tumor volume in each group: in comparison to the PBS group, the tumors in the HPB + NIR group and the CaO_2_ + NIR group continued to grow uncontrollably. However, the DOX + NIR group showed only a mild inhibition of tumor growth, whereas the PCDP and PCDP + NIR groups significantly inhibited tumor growth (Fig. 8D). Further H&E results from tumor slices demonstrated the pathological alterations at the tumor location following therapy. Nearly all cancer cells in the PBS + NIR and HPB + NIR groups retained cell integrity morphology, however a significant proportion of cancer cell structures were destroyed in the PCDP group and PCDP + NIR group, demonstrating that nano-catalysts had a significant impact on cell death (Fig. S8).

Apart from their therapeutic effects, the biosafety of nano-catalysts should also be considered. On the 14th day, we performed H&E staining on the primary organs of nude mice and found no significant histopathological damage in major organs (Fig. S9). It is worth noting that none of the mice in any of the groups experienced a substantial weight loss (Fig. 8E). These results demonstrated the excellent biocompatibility and biosafety of PCDP, which may be attributed to the strong biocompatibility of PAA and the low cytotoxicity of HPB.

These results indicate that, both in terms of tumor cell proliferation rate and cell death rate, the triple therapy demonstrates superior therapeutic efficacy compared to individual or dual treatments. This may be closely associated with the activation of multiple apoptotic pathways within the cells.

## Conclusions

In summary, the PCDP nano-catalyst has shown great potential in achieving TME-responsive CDT/PTT/CT synergistic cancer therapy. The in vitro and in vivo results demonstrated that the nano-catalyst can produce synergistic antitumor effects while maintaining excellent biocompatibility. Therefore, the PCDP nano-catalyst is a highly promising candidate for synergistic cancer therapy. Further studies on optimizing the target, dosage, administration route, and combination with other therapies are necessary to fully evaluate its potential for clinical translation. Nevertheless, the current findings provide valuable insights into the development of highly effective and safe cancer treatments.

### Supplementary Information

Supplementary Figures.

## Data Availability

All data analyzed during this study are included in this published article.
