# Supplementary material for: A TME-activated nano-catalyst for triple synergistic therapy of colorectal cancer
Source: Sci Rep. 2024 Feb 9;14:3328. doi: 10.1038/s41598-024-53334-3 (PMC10858196; doi:10.1038/s41598-024-53334-3)
Supplement: Supplementary file 1 — Supplementary Figures. [file 41598_2024_53334_MOESM1_ESM.docx]

**Supporting Information**

**A TME‑Activated nano-catalyst for Triple Synergistic Therapy of Colorectal Cancer.**

Qiang Liu^1 2 3#^, Yurong Xiang^1 2#^, Qiang Yu^1 2^, Quan Lv^1 2^, Zheng Xiang^1 2*^

*1. Department of General Surgery, The First Affiliated Hospital of Chongqing Medical University Chongqing, China.*

*2. Chongqing Key Laboratory of Department of General Surgery, The First Affiliated Hospital of Chongqing Medical University Chongqing, China.*

*3. Department of Hepatobiliary Surgery, Suining First People's Hospital, Suining, China.*

Here, more information on sample characterization is provided, including the hydration diameter of the sample, Zeta potentials, element analysis, etc. In addition, there are Bio-TEM images, Fluorescence images of live/dead staining, H&E stabilized images, and so on.


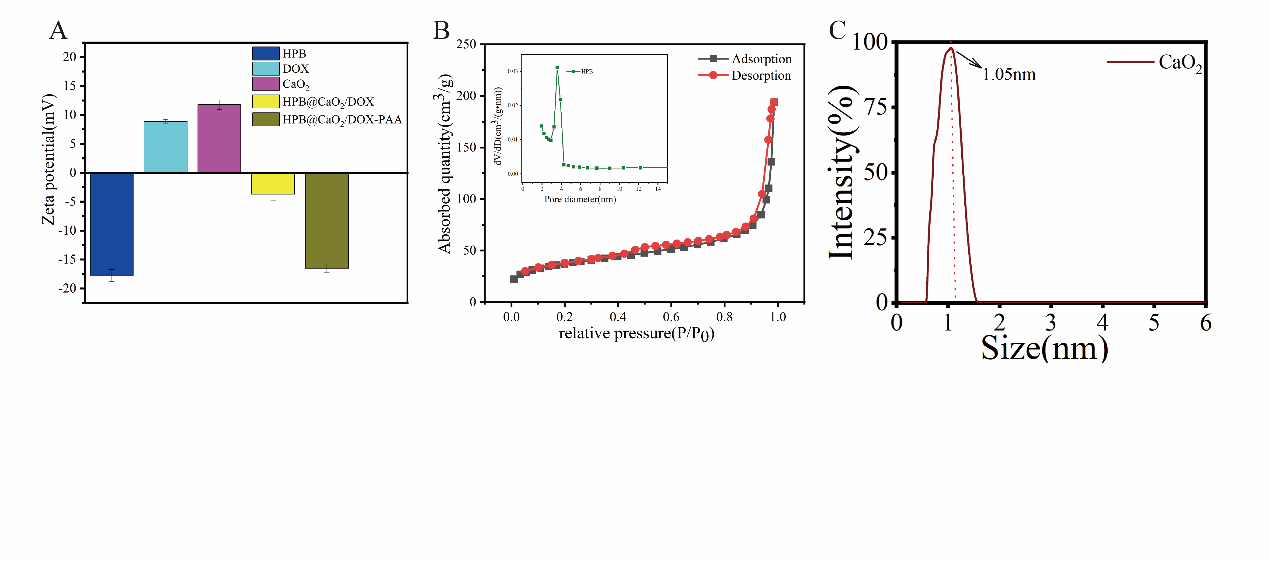


Figure S1. (A) Zeta potentials of HPB、DOX、CaO_2_、HPB@CaO_2_/DOX and [HPB@CaO_2_/DOX-PAA. (B)](mailto:HPB@CaO2/DOX-PAA.(B)) N_2_ adsorption-desorption isotherm of HPB, inset: the corresponding pore size distribution. (C) Pore size distribution of CaO_2_.


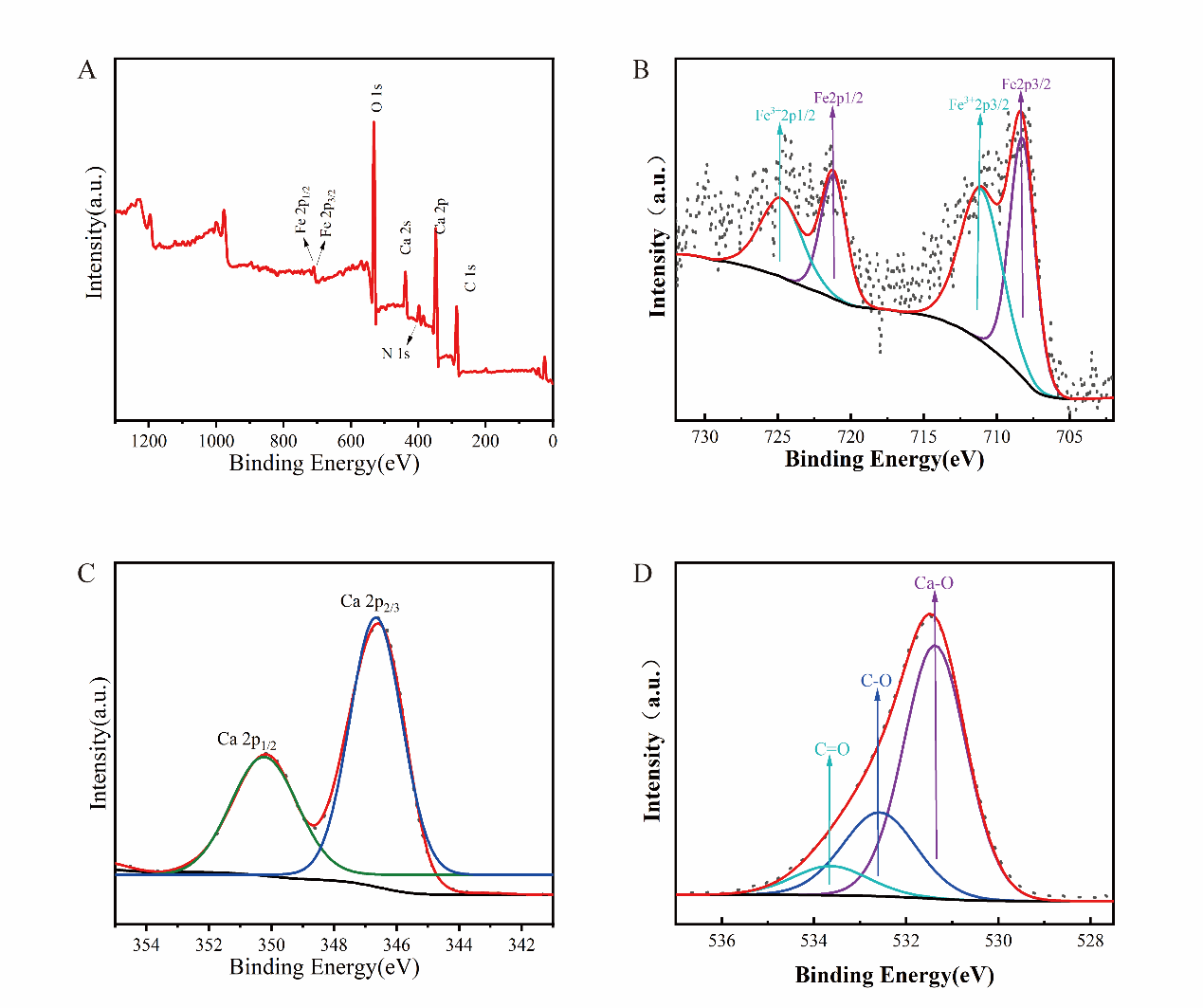


Figure S2 XPS spectrum of HPB@CaO_2_/DOX-PAA: full-survey spectrum (A), Ca 2p spectrum (C), O 1s spectrum (D), and Fe 2p spectrum(B).


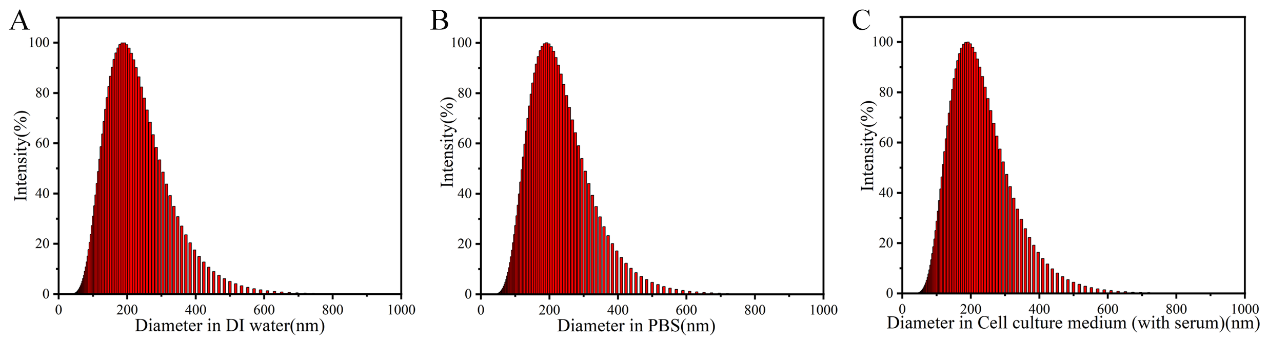


Figure S3. Characterization of the particle size of HPB@CaO_2_/DOX-PAA nanoparticles after standing for 3 days with different components (DI water, PBS (pH7.4), culture medium (with serum)).


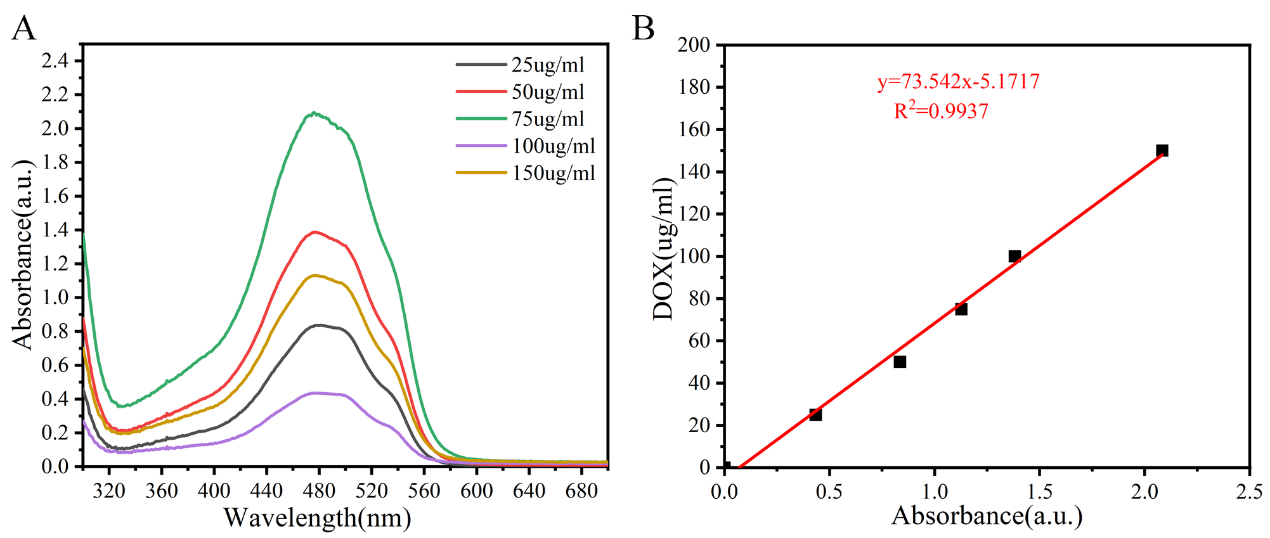


Figure S4. Absorbance at 480nm between DOX concentration(A) and the linear relationship (B).


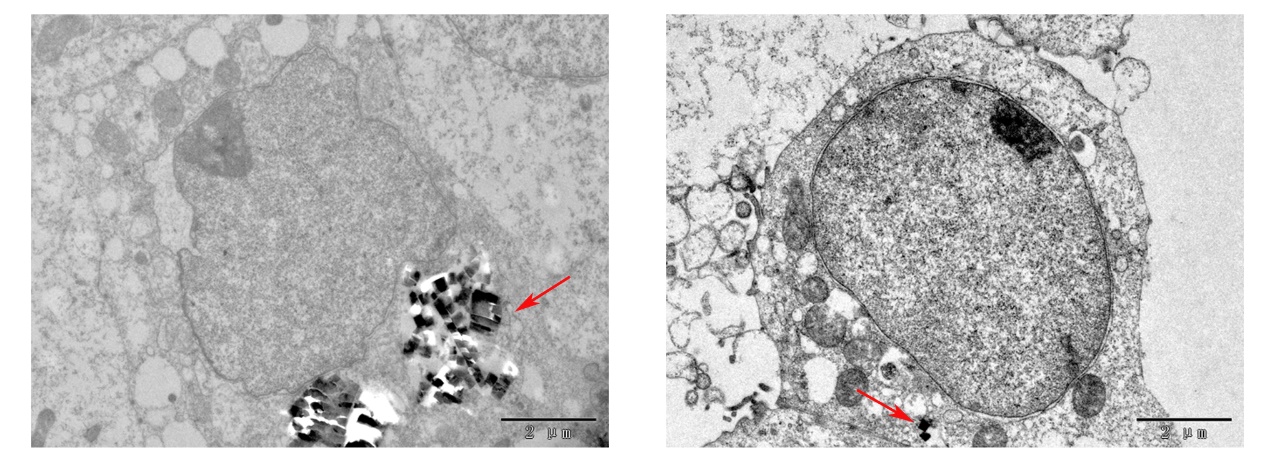


Figure S5. Bio-TEM images of Caco-2 cells after incubation with PCDP for 4 h.


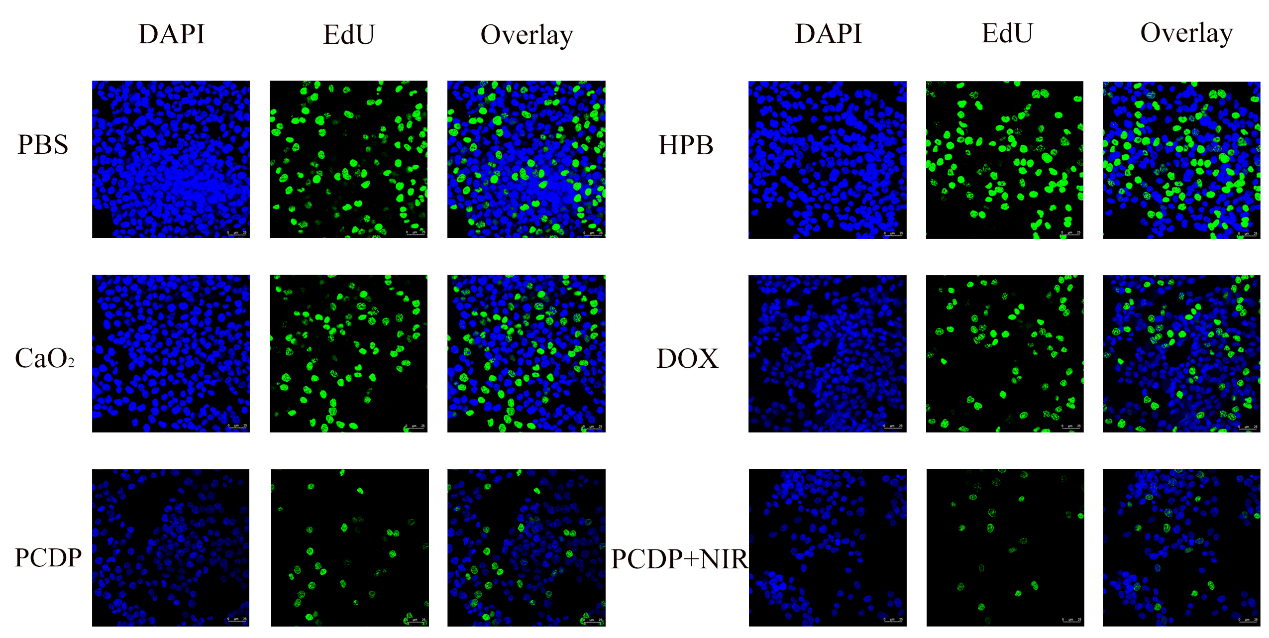


Figure S6. CLSM images of EdU cell proliferation staining in Caco-2 cells after treatments with PBS、HPB、CaO2、DOX、PCDP and PCDP+NIR, respectively. Cell nucleus (blue), proliferating cell (green), Scale bar:25μm.


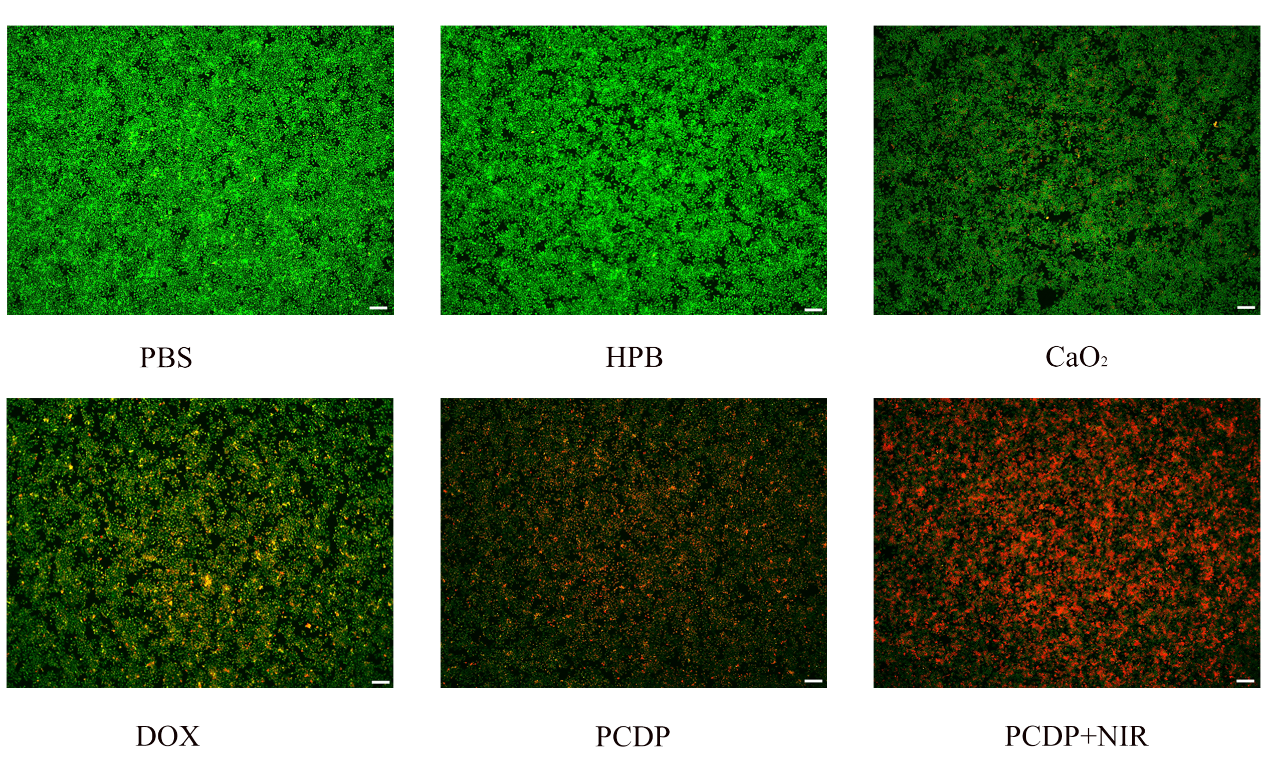


Figure S7. Fluorescence images of live/dead staining in Caco-2 cells. Live cells (green), dead cells (red). After treatmented with PBS, HPB, CaO_2_, DOX, PCDP and PCDP+NIR in Caco-2 cells. Scale bar: 50μm.
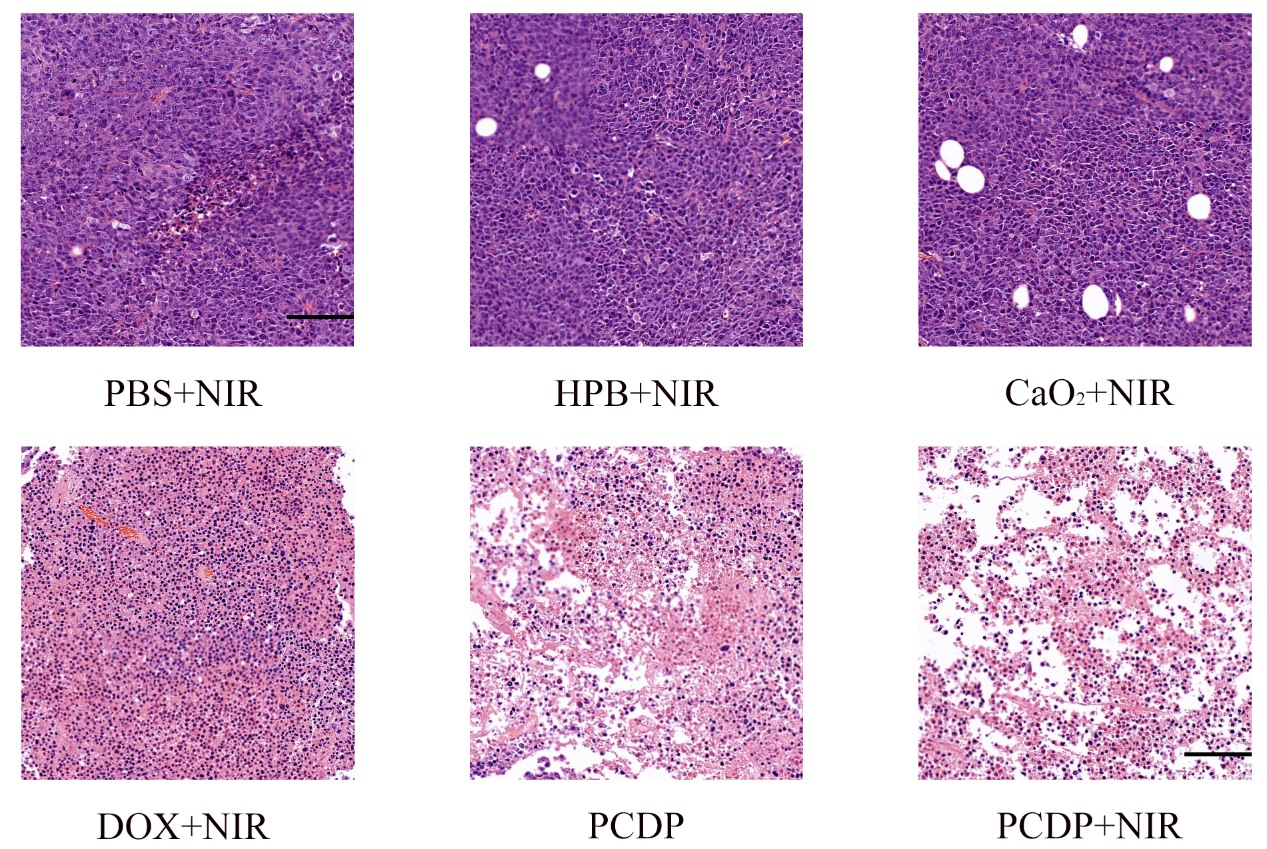


Figure S8. H&E staining of tumor tissues with different treatments. Scale bar, 100μm.


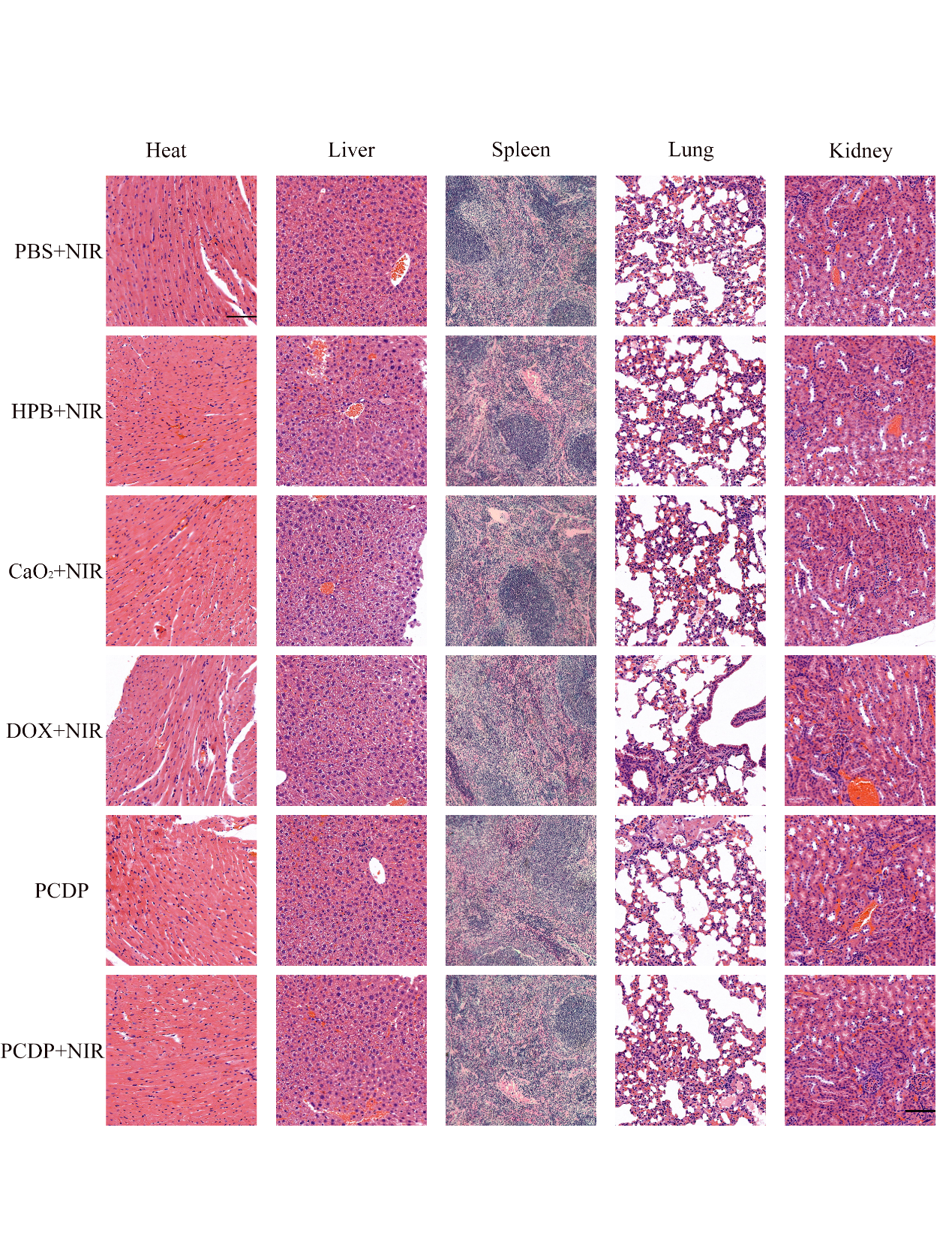


Figure S9. H&E staining of the heart, liver, spleen, lung, kidney with different treatments. Scale bar, 100μm.
